# Vitamin D, vitamin B12, folate, homocysteine, and major haemato-chemical parameters in patients with mood disorders

**DOI:** 10.1192/j.eurpsy.2024.1399

**Published:** 2024-08-27

**Authors:** D. Marazziti, R. Gurrieri, F. Mucci

**Affiliations:** ^1^Department of Clinical and Experimental Medicine, Section of Psychiatry; ^2^Department of Surgical-Medical and Molecular Pathology and Critical Care Medicine, University of Pisa, Pisa, Italy

## Abstract

**Introduction:**

The potential involvement of the immune and inflammatory systems has been extensively studied in mood disorders (MDs). Despite these findings and despite the fact that the pathogenetic role of altered immunologic and metabolic profiles in MDs is being confirmed in many current studies, there is still a lack of consensus about it, due to controversial results.

**Objectives:**

The present study aimed to appraise peripheral metabolic parameters (blood glucose, lipoproteins, triglycerides, uric acid, blood urea nitroge [BUN], transaminases and others9 and plasma/serum levels of essential nutrients (vitamin D, B12, folate and homocysteine) in a group of inpatients affected by MDs, as compared with healthy controls.

**Methods:**

Methods. Ten ml of venous blood was drawn from fasting subjects. The metabolic parameters and vitamins were measured according to common clinical-chemistry methods.Comparisons for continuous variables were performed by the Student’s t-test for variables that follow a normal distribution, and by the Wilcoxon-Mann-Whitney test for variables not normally distributed. The correlations between biological markers were explored by calculating the Pearson’s correlation coefficient or Spearman rank correlation.

**Results:**

Most patients showed loer circulating vitamin D levels, in respect to both control subjects (P< .0001) and the normative cut-off values. This finding was paralleled by increased serum homocysteine concentrations i (P<.0001), indicating an imbalance in their methionine metabolism. Homocysteine levels were negatively correlated with vitamin D, vitamin B12 and folate in control subjects, but not in patients. In addition, patients displayed higher blood glucose and lower BUN than controls, indicating an impaired protein-to-carbohydrate metabolism and/or altered nutritional/dietary status.

**Conclusions:**

We provide herein further support to the notion that MD patients are a population where vitamin deficits, dysmetabolism and/or dietary defects are common feature, and, s such, they might be more vulnerable to a variety of somatic illnesses than the general population. This cross-sectional investigation, albeit preliminary, might contribute to improve the characterization and the monitoring of the clinical status of mood disorder patients, as well as to identify new molecular targets for more tailored treatments ad of more pointed health-care intervention,

**Disclosure of Interest:**

None Declared

